# Punicalagin Alleviates Psoriasis by Inhibiting NF-κB-Mediated IL-1β Transcription and Caspase-1-Regulated IL-1β Secretion

**DOI:** 10.3389/fphar.2022.817526

**Published:** 2022-01-26

**Authors:** Lipeng Tang, Tong Li, Bowen Zhang, Zihao Zhang, Xiaoyi Sun, Ying Zhu, Bing Feng, Zuqing Su, Laijun Yang, Hongxia Li, Huazhen Liu, Yuchao Chen, Zhenhua Dai, Xirun Zheng, Mingxian Li, Chutian Li, Jie Zhao, Xinmin Qiu, Shuyan Ye, Han Liu, Guangjuan Zheng, Ben Li, Chuanjian Lu

**Affiliations:** ^1^ State Key Laboratory of Dampness Syndrome of Chinese Medicine, The Second Clinical College of Guangzhou University of Chinese Medicine, Guangzhou, China; ^2^ Guangdong-Hong Kong-Macau Joint Lab on Chinese Medicine and Immune Disease Research, The Second Clinical College of Guangzhou University of Chinese Medicine, Guangzhou, China; ^3^ Department of Pharmacology of Traditional Chinese Medicine, The Second Clinical College of Guangzhou University of Chinese Medicine, Guangzhou, China; ^4^ Department of Pharmacy, The Second Clinical College of Guangzhou University of Chinese Medicine, Guangzhou, China; ^5^ School of Life Sciences and Biopharmaceutics, Guangdong Pharmaceutical University, Guangzhou, China; ^6^ Section of Immunology, Guangdong Provincial Academy of Chinese Medical Sciences, Guangzhou, China; ^7^ Department of Pathology, The Second Clinical College of Guangzhou University of Chinese Medicine, Guangzhou, China; ^8^ Genetic Testing Lab, The Second Clinical College of Guangzhou University of Chinese Medicine, Guangzhou, China; ^9^ Department of Dermatology, The Second Clinical College of Guangzhou University of Chinese Medicine, Guangzhou, China; ^10^ Guangdong Provincial Key Laboratory of Clinical Research on Traditional Chinese Medicine Syndrome, The Second Clinical College of Guangzhou University of Chinese Medicine, Guangzhou, China

**Keywords:** psoriasis, punicalagin, NF-κB, caspase-1, IL-1β

## Abstract

Psoriasis is a chronic and inflammatory skin disorder characterized by inflammation and epidermal hyperplasia. Punicalagin (PUN) is a main active ingredient of pomegranate (*Punica granatum* L.) peel with multiple biological activities, such as antibacterial, antioxidant and anti-tumor effects. However, the potential effect of PUN on psoriasis remains unknown. In this study, we want to investigate the pharmacological effect of PUN on psoriasis by using imiquimod (IMQ)-induced psoriatic mice model *in vivo* and tumor necrosis factor *a* (TNF-α) and interleukin-17A (IL-17A)-stimulated HaCaT cells *in vitro*. Our results showed that PUN can effectively alleviate the severity of psoriasis-like symptoms. Mechanistically, PUN potently suppresses the aberrant upregulation of interleukin-1β (IL-1β) and subsequent IL-1β-mediated inflammatory cascade in keratinocytes by inhibiting the nuclear factor kappa B (NF-κB) activation and cleaved caspase-1 expression *in vitro* and *in vivo*. Taken together, our findings indicate that PUN can relieve psoriasis by repressing NF-κB-mediated IL-1β transcription and caspase-1-regulated IL-1β secretion, which provide evidence that PUN might represent a novel and promising candidate for the treatment of psoriasis.

## Introduction

Psoriasis is a chronic, recurrent, inflammatory skin disease affecting more than 60 million people worldwide (Parisi et al., 2013). The typical clinical manifestations of psoriasis are abnormal papules, erythema, thickened scaly spots, local dull pain and skin itching (Nestle et al., 2009), which seriously affect the patient’s quality of life. Despite the newly developed biologics, such as adalimumab, guselkumab and mirikizumab (Armstrong and Read, 2020; Ghoreschi et al., 2021), have revolutionized moderate-to-severe plaque psoriasis, the biologics are too expensive for ordinary patients. Additionally, the long-term use of the biologics may lead to serious side effects (Scherer et al., 2010; Reich et al., 2015), including tumors. In addition, mild-to-moderate plaque psoriatic patients are usually treated with topical therapies (glucocorticoids and vitamin D derivatives) or systemic medications (methotrexate, acitretin, and cyclosporine) (Armstrong and Read, 2020). However, topical therapies or systemic medications have many contraindications or side effects (Boehncke and Schön, 2015; Reich et al., 2015). Therefore, seeking an economical, safe and effective treatment is extremely urgent for psoriasis.

Although most psoriasis-related researches have mainly focused on the interleukin-23 (IL-23)/interleukin-17 (IL-17) axis, emerging evidence indicates that interleukin-1β (IL-1β) also plays a critical role in psoriasis. Moorchung et al. (2015) identified a strong association of IL-1β *511C/T* (the C/T base transitions at positions -511 in the transcription initiation site) polymorphism with the genotypes and alleles in psoriasis. Moreover, Hébert et al. (2014) showed that the polymorphism of IL-1β *gene* could help to discriminate the early or late onset of psoriasis. Additionally, several studies indicated that IL-1β expression was significantly increased in both psoriatic plasma and skin lesion (Cai et al., 2019b; Verma et al., 2021). Furthermore, upregulated IL-1β could further amplify the skin inflammation by enhancing IL-17 production in dermal γδ T-cell and promoting chemokines [such as C-X-C motif chemokine ligand 1 (CXCL-1) and C-C motif chemokine ligand 20 (CCL-20)] secretion in keratinocytes (Cai et al., 2011; Cai et al., 2019b). Interestingly, IL-1β has also been pointed to be involved in the recurrence of psoriasis. Naik et al. (2017) showed that epithelial stem cells (EpSCs) in the psoriatic skin lesions can release numerous IL-1β upon a secondary stimulation, ultimately leading to inflammation recollection and psoriasis recurrence. Taken together, these findings suggest that IL-1β might be a promising therapeutic target for psoriasis intervention.

Punicalagin (PUN, Supplementary Figure S1), which is the main active ingredient of pomegranate (*Punica granatum* L.) peel, possesses multiple bioactivities, such as antioxidant (Chen et al., 2012), antibacterial (Sun Q. et al., 2019) and anti-tumor (Morsy et al., 2019; Huang et al., 2020; Pan et al., 2020) activities. Intriguingly, the regulatory effects of punicalagin on suppressing inflammation have attracted the researcher’s attention. Punicalagin is proved to be able to alleviate various inflammatory diseases, including inflammatory bowel disease (Shah et al., 2016), ankylosing spondylitis (Feng et al., 2020) and rheumatoid arthritis (Huang et al., 2021). However, it is still unknown whether PUN exert a possible therapeutic effect on inflammatory skin disease, in particular psoriasis.

To evaluate the potential pharmacological effect of PUN on psoriasis, we made use of imiquimod (IMQ)-induced psoriasis-like mice model and tumor necrosis factor α (TNF-α) and IL-17A-stimulated HaCaT cells. We found that PUN can relieve psoriasis-like symptoms by suppressing the nuclear factor kappa B (NF-κB)-mediated IL-1β transcription and caspase-1-regulated IL-1β secretion *in vivo* and *in vitro*. Our findings suggest that PUN may represent a promising lead compound for developing newly anti-psoriatic drugs.

## Materials and Methods

### Experimental Reagents

Punicalagin (P0023, purity ≥98%) was purchased from Sigma-Aldrich (St. Louis, MO, United States). IMQ cream was obtained from Sichuan Med-Shine Pharmaceutical Co., Ltd. (Sichuan, China). IL-17A (12047-HNAE), TNF-α (10602-HNAE), IL-1β (10139-HNAE) were purchased from Sino Biological Inc. (Beijing, China).

### Mice and its Experiments

Male BALB/c mice (6–8 weeks of age), which were purchased from Animal Research Laboratory of Guangdong Province (Guangzhou, China), were maintained under specific pathogen-free conditions. After quarantine, mice were divided into five groups: Control group, IMQ group, IMQ + DEX group (DEX, dexamethasone cream, positive drug for psoriasis), IMQ + Vehicle group, IMQ + PUN group (25 mg/kg PUN). The shaved back of mice was applied with a daily topical dose of 62.5 mg of IMQ cream (5%) at 9:00 am for 7 consecutive days. In addition, control mice were treated with the same dose of Vaseline. Moreover, mice in IMQ + DEX group were topically administrated with DEX once a day (at 12:00 am) for 7 consecutive days. Furthermore, mice in IMQ + Vehicle group or IMQ + PUN group were topically administrated with vehicle gel (Sun et al., 2013) (0.3 g carbomer 940, 0.15 g azone, 4.5 g 96% ethanol and distilled water to 15 g) or PUN gel (0.3 g carbomer 940, 0.15 g azone, 4.5 g 96% ethanol, 78.125 mg PUN powder and distilled water to 15 g) twice a day (at 12:00 am and 4:00 pm) for 7 consecutive days. This experiment is finished on the eighth day, and follow-up verification is carried out. All procedures were approved and supervised by Guangzhou University of Chinese Medicine Animal Care and Use Committee (Approval No. 2020082, Guangdong, China).

### Hematoxylin and Eosin Staining

The experiment was terminated on the eighth day. The skin collected from mice was fixed in neutral formalin for 48 h, then dehydrated and embedded in paraffin. Slicing the cross-section of the skin, the morphological characteristics of different groups mice were observed under a microscope (Olympus) after H&E staining. Acanthosis hyperplasia is evaluated by Photoshop 2021 software, the cortical direction of all pictures is adjusted to the level and the size of 1,000 pixels * 1,000 pixels is intercepted, finally the lasso tool is used to determine the pixels of the acanthosis layer in this size. Acanthosis (in^2^) = width pixels * height pixels/horizontal resolution * vertical resolution.

### Immunohistochemical Staining

Immunohistochemical staining was performed using paraffin-embedded mouse back skin sections. Experiment was operated in the following order: baking slices, dewaxing, antigen retrieval and membrane rupture. After this, the samples were stained with 5% bovine serum albumin (BSA) for 1 h at room temperature, Ki67 antibody (16667, Abcam) or phospho-NF-κB (Ser536) p65 (AF2006, Affinity) incubation for overnight, and further incubated with the anti-rabbit antibody. Using diaminobenzidine (DAB) staining solution to color development and hematoxylin to counterstain the nucleic acids. Dehydrated to wax before observed under the Olympus microscope. Ki67 positive cells per 10 cm are the number of Ki67 positive staining cells in the area of 10 * 10 cm at random, and three areas are selected for each picture. Expression of phosphorylated p65 per 10 cm is the number of cells expressed phosphorylated p65 (Ser536) in the area of 10 * 10 cm at random, and three areas are selected for each picture.

### Cell Culture and Stimulation

Human HaCaT cells (human keratinocytes) were purchased from China Center for Type Culture Collection (GDC106) and examined without pollution. HaCaT cells were cultured in MEM α Nucleosides Medium (C12571500BT, Gibco) containing 10% fetal bovine serum (FBS) (10,099-141C, Gibco), 1% Sodium Pyruvate (100X) (11360070, Gibco) and 1% MEM Non-Essential Amino Acids Solution (100X) (11140050, Gibco) in 37° and 5% CO_2_ incubator.

### Quantitative real-Time PCR

The whole operations were performed in an RNA-free environment and used RNA-free tools. First, total RNA was extracted from cell or skin samples using TRIzol™ reagent (15596018, Life Technologies). Then, 1 μg RNA was used for reverse transcription by using a cDNA Reverse transcriptase kit (K1622, Thermo Scientific). The qPCR was performed in triplicate using SYBR green master mix (FP205-02, Tiangen) on Real-Time PCR System (Applied Biosystem 7,500, Life Technologies). *GAPDH* was used as the reference gene for normalization.

### Western Blotting

Total protein was extracted from samples using RIPA buffer (P0013C, Beyotime) containing 1% phosphatase and protease inhibitors (P1206, Applygen) following the instructions. The nuclear and cytoplasm protein fractions were isolated using the NE-PER Nuclear and Cytoplasmic Extraction Reagents (78,835, Thermo Scientific) following the instructions. Using BCA Protein Assay Kit (9S8K-29538-413, Thermo Scientific) normalized the total protein for the follow-up experiments. Quantified samples were separated by sodium dodecyl sulfate-polyacrylamide gel electrophoresis (SDS-PAGE) and transferred to polyvinylidene fluoride (PVDF) membranes (IPYH00010, Merck Millipore). 5% skimmed milk is slowly shaken at room temperature to block the membrane for 1 h and then primary antibodies incubate these membranes overnight at 4°C. The next day membranes were incubated by the species-specific secondary antibody at room temperature for 1 h and exposed by Immobilon Western Chemiluminescent HRP Substrate (WBKLS0500, Merck Millipore). The antibodies used in the experiment are as follows: GAPDH (1:2,000; 60004-1-Ig, Proteintech), *ß*-Tubulin (1:2,000; 10094-1-AP, Proteintech), Lamin B1 (1:2,000; 12987-1-AP, Proteintech), IL-1β (1:1,000; 216,995, Abcam), cleaved IL-1β (1:1,000; 83,186, CST), NF-κB p65 (1:1,000; 8,242, CST), phospho-NF-κB p65 (Ser536) (1:1,000; 3,033, CST), cleaved caspase-1 (1:1,000; 4,199, CST) and caspase-1 (1:1,000; 179,515, Abcam) for HaCaT cells samples; IL-1β (1:1,000; 234,437, Abcam), cleaved IL-1β (1:1,000; 63,124, CST), caspase-1 (1:1,000; 24,232, CST) and cleaved caspase-1 (1:1,000; 89,332, CST) for mice samples. GAPDH and *ß*-Tubulin were shown as housekeeping proteins for the normalization of total protein or cytoplasmic protein; Lamin B1 as the loading control for nuclear protein.

### Immunofluorescence Staining

HaCaT cells were seeded and treated in the 96-well plates. After being fixed in 4% paraformaldehyde, the HaCaT cells were permeabilized with phosphate buffer solution (PBS) containing 0.5% Triton X-100 for 10 min, and subsequently were blocked with 5% BSA for 1 h. Next, the primary antibody against p65 (1:1,200; 8,242, CST) incubated the cells overnight at 4°C. Then anti-rabbit IgG (H + L), F(ab')2 fragment secondary antibody (1:500; 4,413, CST) was used to incubate cells for 60 min at room temperature. After counterstaining with 4′, 6-diamidino-2-phenylindole (DAPI), the cell in the 96-well plates were observed and imaged under the Nikon microscope.

### Statistical Analysis

Statistical analysis was performed using SPSS and Sigmaplot software. The whole statistics were presented as mean ± standard error of mean (S.E.M.). Analysis of one-way ANOVA with Student-Newman-Keuls method or non-parametric datasets with Kruskal–Wallis test was used for multiple comparisons. Probability values <0.05 were considered significant. *p < 0.05; **p < 0.01; ***p < 0.001; *N.S.*, non-significant. Error bars depict S.E.M.

## Results

### Punicalagin Alleviates Psoriasis-Like Symptoms in IMQ-Induced Mice

**FIGURE 1:**
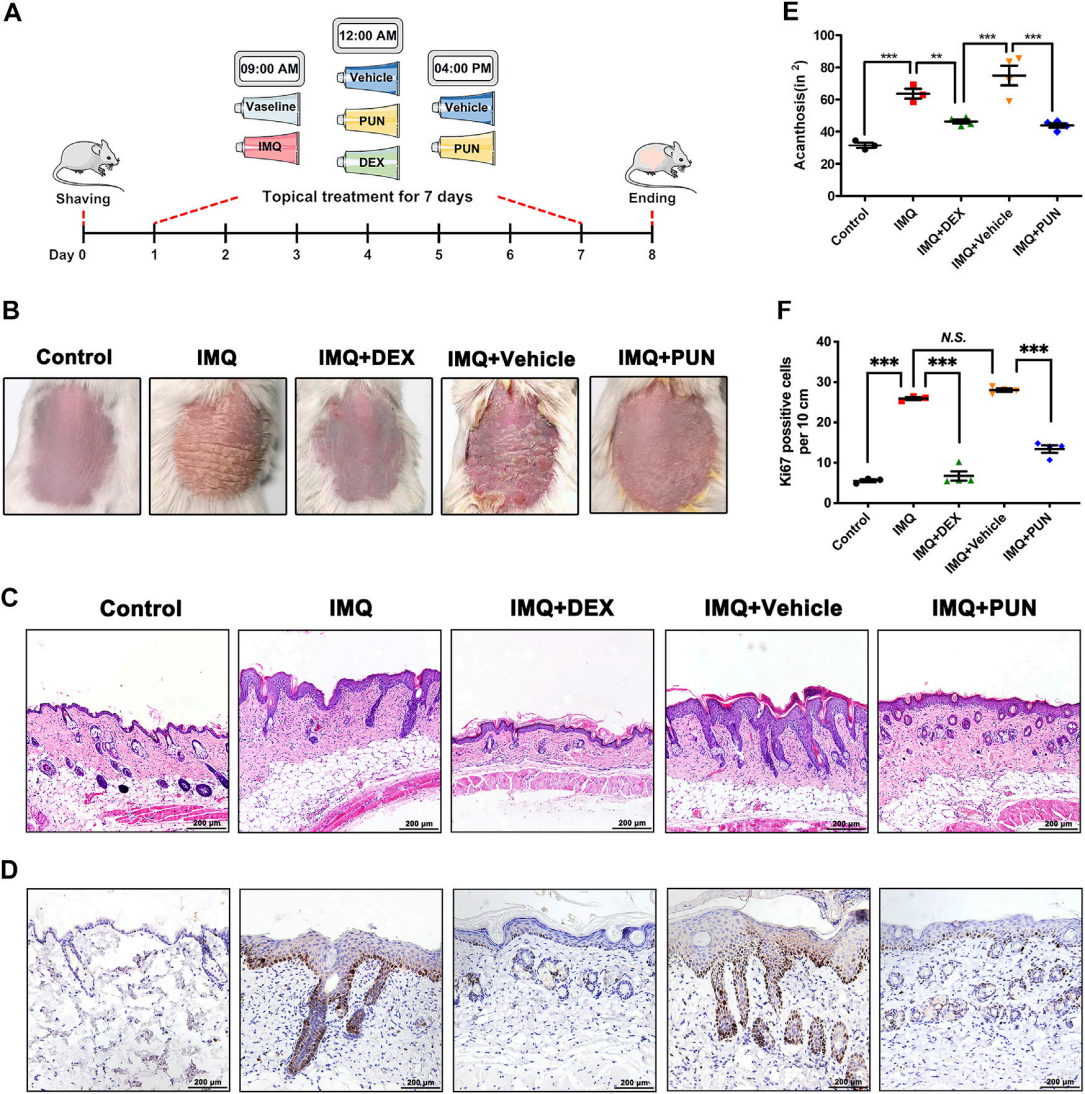
Punicalagin significantly ameliorates IMQ-induced psoriasis-like symptoms *in vivo*. **(A)** A schematic showing the experimental design. **(B)** Phenotypic appearances of mice back skin in Control/IMQ/IMQ + DEX/IMQ + Vehicle/IMQ + PUN groups on day 8. **(C)** Histological changes of mice back skin in Control/IMQ/IMQ + DEX/IMQ + Vehicle/IMQ + PUN groups. Scale bar, 200 μm. **(D)** Ki67 staining of mice back skin in Control/IMQ/IMQ + DEX/IMQ + Vehicle/IMQ + PUN groups. Scale bar, 200 μm. **(E)** Histological analysis of acanthosis in Control/IMQ/IMQ + DEX/IMQ + Vehicle/IMQ + PUN groups. **(F)** The numbers of Ki67 positive cells in lesion skin derived from mice treated with Vaseline/IMQ/IMQ + DEX/IMQ + Vehicle/IMQ + PUN. **p < 0.01, ***p < 0.001, *N.S.*, non-significant. One-way ANOVA with Student-Newman-Keuls method for **(E, F)**. Data represent the mean ± S.E.M.

To evaluate whether PUN has potential pharmacological effects on psoriasis, we conducted an IMQ-induced psoriatic *in vivo* model and then topically applied with DEX cream, vehicle gel or PUN gel on the shaven back skin of IMQ-treated mice for consecutive 7 days (Figure 1A). We found that IMQ treatment can cause severe psoriasis-like phenotypes, including erythema and severe plaque. In addition, mice with vehicle gel treatment also developed psoriasis-like manifestations. However, these psoriatic-related phenotypes can be improved by application of DEX or PUN (25 mg/kg) (Figure 1B). The results of H&E staining and histological analysis were in line with the macroscopic appearance. Mice treating with IMQ or IMQ + Vehicle gel became severe epidermal hyperplasia and acanthosis. As expected, topical administration of DEX attenuated IMQ-induced epidermal thickening and acanthosis. Similarly, topical treatment with 25 mg/kg PUN also ameliorated the above-mentioned manifestations (Figures 1C,E). Moreover, we also detected the expression of Ki67. We found that Ki67-positive cells were significantly reduced in the skin lesions after application of DEX or PUN gel (Figures 1D,F). Taken together, these results indicate that PUN can effectively relieve the severity of IMQ-induced psoriatic-like symptoms.

### Punicalagin Reduces TNF-α and IL-17A-Induced IL-1β Upregulation *via* Inhibiting the Activation of NF-κB and the Expression of Caspase-1 *in Vitro*

**FIGURE 2:**
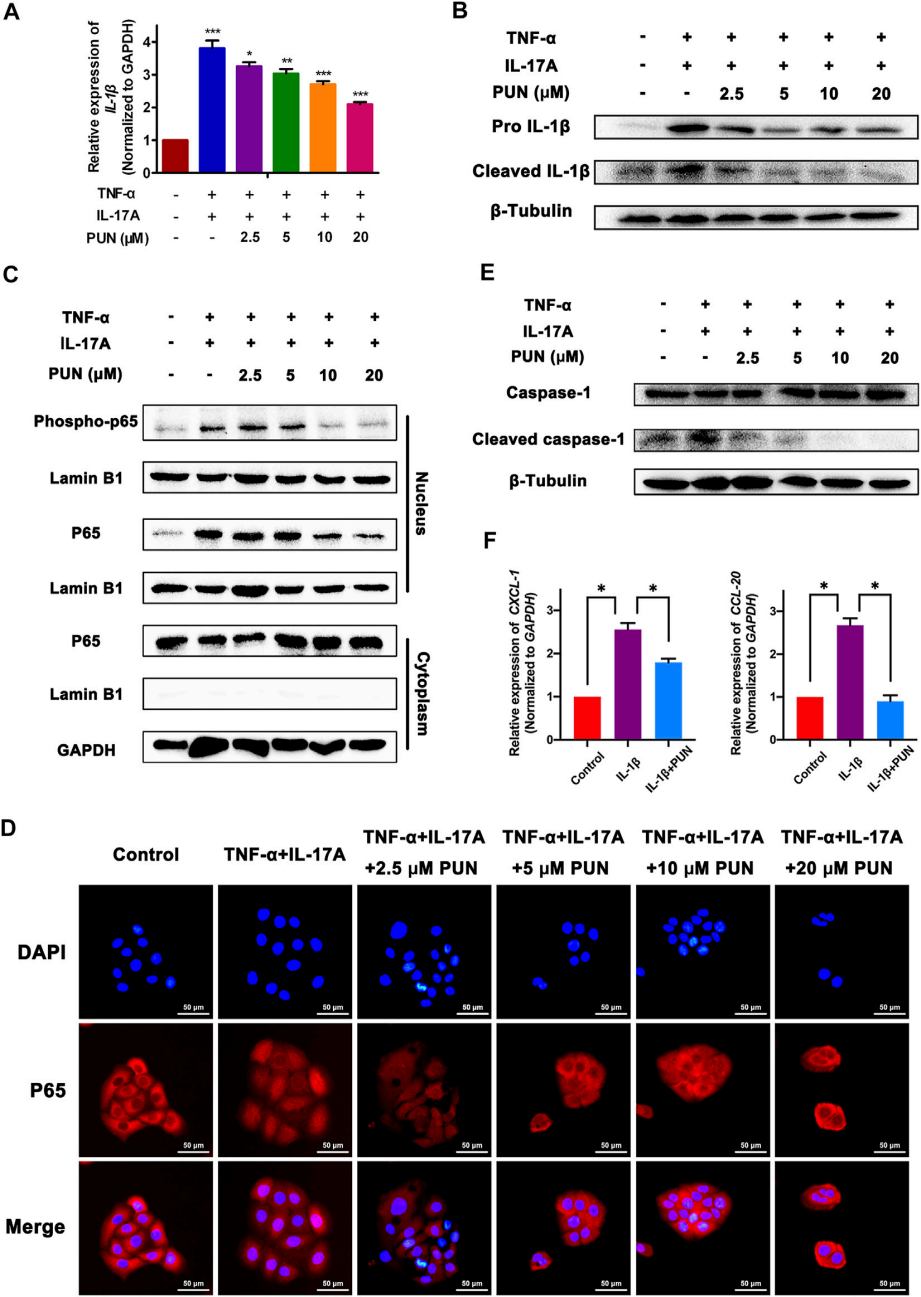
Punicalagin suppresses the TNF-α and IL-17A-induced IL-1β upregulation *via* inhibiting NF-κB activation and cleaved caspase-1 expression *in vitro*. **(A)** The relative mRNA level of *IL-1*β in different groups. Here we used IL-17A (25 ng/ml) and TNF-α (25 ng/ml) to stimulate HaCaT cells. Simultaneously, we also added 2.5/5/10/20 μM PUN into these IL-17A and TNF-α-stimulated HaCaT cells. The total RNA was subsequently extracted after treatment with IL-17A (25 ng/ml) + TNF-α (25 ng/ml) and 2.5/5/10/20 μM PUN for 24 h. **(B)** The pro- and mature expression of IL-1β in different groups. The total protein was collected after treatment with IL-17A (25 ng/ml) + TNF-α (25 ng/ml) and 2.5/5/10/20 μM PUN for 48 h. **(C)** The expression of phosphorylation (Ser536) and total p65 in the cytoplasm and nucleus after exposure of IL-17A (25 ng/ml) + TNF-α (25 ng/ml) and 2.5/5/10/20 μM PUN for 24 h. **(D)** Immunostaining with an anti-p65 antibody showed that 2.5/5/10/20 μM PUN functionally blocks TNF-α and IL-17A-induced nuclear translocation of p65. Scale bar, 50 μm. **(E)** The expression of cleaved and total caspase-1 after exposure of IL-17A (25 ng/ml) + TNF-α (25 ng/ml) and 2.5/5/10/20 μM PUN for 48 h. **(F)** The relative mRNA level of *CXCL-1* (left) and *CCL-20* (right) in HaCaT cells after treatment with IL-1β and IL-1β + PUN for 48 h *p < 0.05, **p < 0.01, ***p < 0.001. One-way ANOVA with Student-Newman-Keuls method for **(A, F)**. Data represent the mean ± S.E.M.

To further explore the underlying mechanism by which PUN ameliorates psoriasis, we tested the anti-inflammatory properties of PUN *in vitro*. Since that IL-17A and TNF-α are the key inflammatory cytokines in the pathogenesis of psoriasis, we used IL-17A and TNF-α to stimulate HaCaT cells and conducted an inflammatory psoriatic *in vitro* model. We found that the mRNA and pro-/mature protein levels of IL-1β, another important cytokine in psoriasis, were significantly increased after stimulation with IL-17A and TNF-α. Intriguingly, PUN can reduce the upregulated IL-1β gene and pro-/mature protein expression in a dose-dependent way (Figures 2A,B). Given the pivotal role of NF-κB pathway in regulating the gene transcription (Xia et al., 2018; Sun P. et al., 2019), we next examined whether PUN can modulate the activation of NF-κB signaling pathway. Our preliminary work found that NF-κB undergoes activation through phosphorylation of p65 (Ser536) and nuclear translocation of p65 (Supplementary Figure S2), which is consistent with the previous studies (Christian et al., 2016; Varshney et al., 2016; Huang et al., 2021). Firstly, we detected the modulatory effects of PUN on the phosphorylation of p65 (Ser536) *in vitro*. We found that the phosphorylation of p65 (Ser536) was markedly enhanced by IL-17A and TNF-α, whereas PUN can functionally reduce the expression of phosphorylation of p65 (Ser536) in a dose-dependent way (Figure 2C). Since activated p65 will perform nuclear translocation and subsequently promote downstream IL-1β gene transcription, therefore, we further examined whether PUN can suppress the nuclear translocation of p65. We found that treatment with PUN (2.5/5/10/20 μM) could effectively block TNF-α and IL-17A-induced nuclear translocation of p65, respectively (Figure 2D). Moreover, pro IL-1β can be cleaved by cleaved caspase-1 and then become mature form of IL-1β. Here we showed that PUN could decrease the enhancement of pro- and mature IL-1β by IL-17A and TNF-α, therefore, we also detected the regulatory effects of PUN on cleaved caspase-1. As shown in Figure 2E, we found that PUN potently reduced the IL-17A and TNF-α-induced upregulation of cleaved caspase-1 in a dose-dependent way. As we all known, excessive IL-1β secretion will fuel psoriatic inflammatory processes by stimulating keratinocytes to secrete multiple chemokines, such as CXCL-1 and CCL-20. Hence, we also detected whether PUN can regulate the IL-1β-mediated secretion of CXCL-1 and CCL-20 in HaCaT cells. The qPCR results revealed that PUN can efficiently decrease the IL-1β-induced *CXCL-1* and *CCL-20* production (Figure 2F). Taken together, our findings suggest that PUN can inhibit the TNF-α and IL-17A-mediated IL-1β upregulation *via* suppressing p65 activation and cleaved caspase-1 expression *in vitro*, finally resulting in the repression of the subsequent IL-1β-mediated chemokines secretion.

### Punicalagin Inhibits NF-κB-Mediated IL-1β Transcription and Caspase-1-Regulated IL-1β Secretion *in Vivo*

**FIGURE 3:**
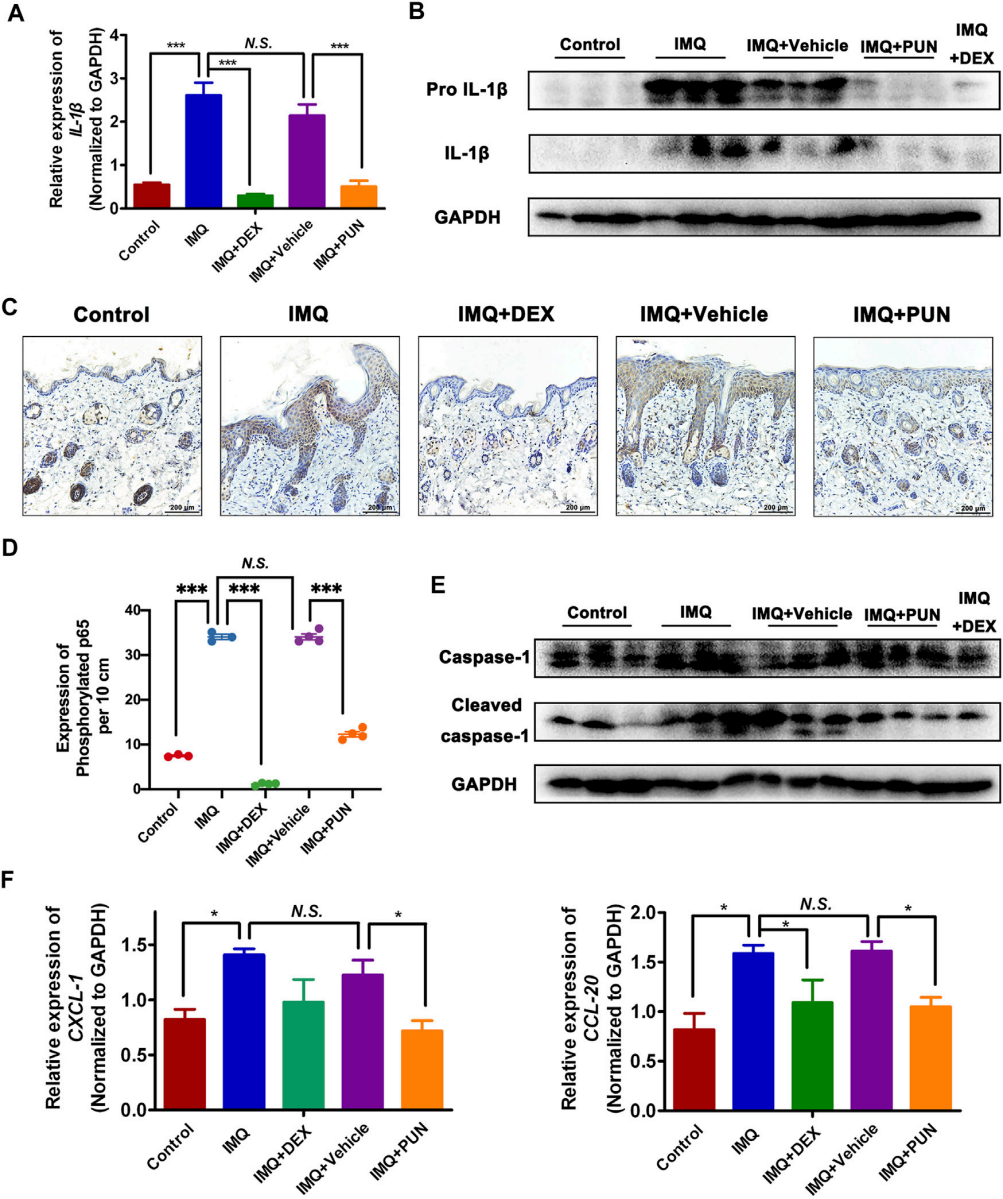
Punicalagin attenuates the enhancement of IL-1β by suppressing NF-κB activation and caspase-1 expression *in vivo*. **(A)** The relative mRNA level of *IL-1*β in Control/IMQ/IMQ + DEX/IMQ + Vehicle/IMQ + PUN groups. **(B)** The protein expression of IL-1β in Control/IMQ/IMQ + DEX/IMQ + Vehicle/IMQ + PUN groups. **(C, D)** Immunohistochemical staining and statistical analysis of phosphorylated p65 (Ser536) in skin sections derived from the Control/IMQ/IMQ + DEX/IMQ + Vehicle/IMQ + PUN group. Scale bars: 200 μm. **(E)** The expression of cleaved and total caspase-1 in skin sections derived from the Control/IMQ/IMQ + DEX/IMQ + Vehicle/IMQ + PUN group. **(F)** The relative mRNA level of *CXCL-1* (left) and *CCL-20* (right) in the skin lesions from Control/IMQ/IMQ + DEX/IMQ + Vehicle/IMQ + PUN groups. *p < 0.05, ***p < 0.001, *N.S.*, non-significant. One-way ANOVA with Student-Newman-Keuls method for **(A, D, F)**. Data represent the mean ± S.E.M.

To determine whether PUN also reduced the IL-1β expression *in vivo*, we measured the gene and protein levels of IL-1β in skin lesion derived from Control/IMQ/IMQ + DEX/IMQ + Vehicle/IMQ + PUN group. As shown in Figures 3A,B, topical application of IMQ or IMQ + Vehicle caused a significant upregulation of IL-1β in both gene and protein level, whereas treatment with PUN potently decreased the upregulated level of IL-1β in IMQ-applied groups. Furthermore, we also examined the potential regulatory effects of PUN on the Ser536 phosphorylated p65 *in vivo*. Consistent with the *in vitro* results, we found that topical administration with IMQ can significantly enhance the levels of phosphorylation of p65 in the keratinocytes. Nonetheless, topical treatment with PUN can remarkably repress the expression of phosphorylated p65 in keratinocytes (Figures 3C,D). Moreover, PUN can also potently suppress the protein expression of cleaved caspase-1 and the mRNA level of *CXCL-1* and *CCL-20 in vivo* (Figures 3E,F). Together, these *in vivo* results also confirm that PUN can functionally attenuate the IL-1β expression and its subsequent inflammatory cascade by repressing the NF-κB activation and the cleaved caspase-1 expression.

## Discussion

**FIGURE 4:**
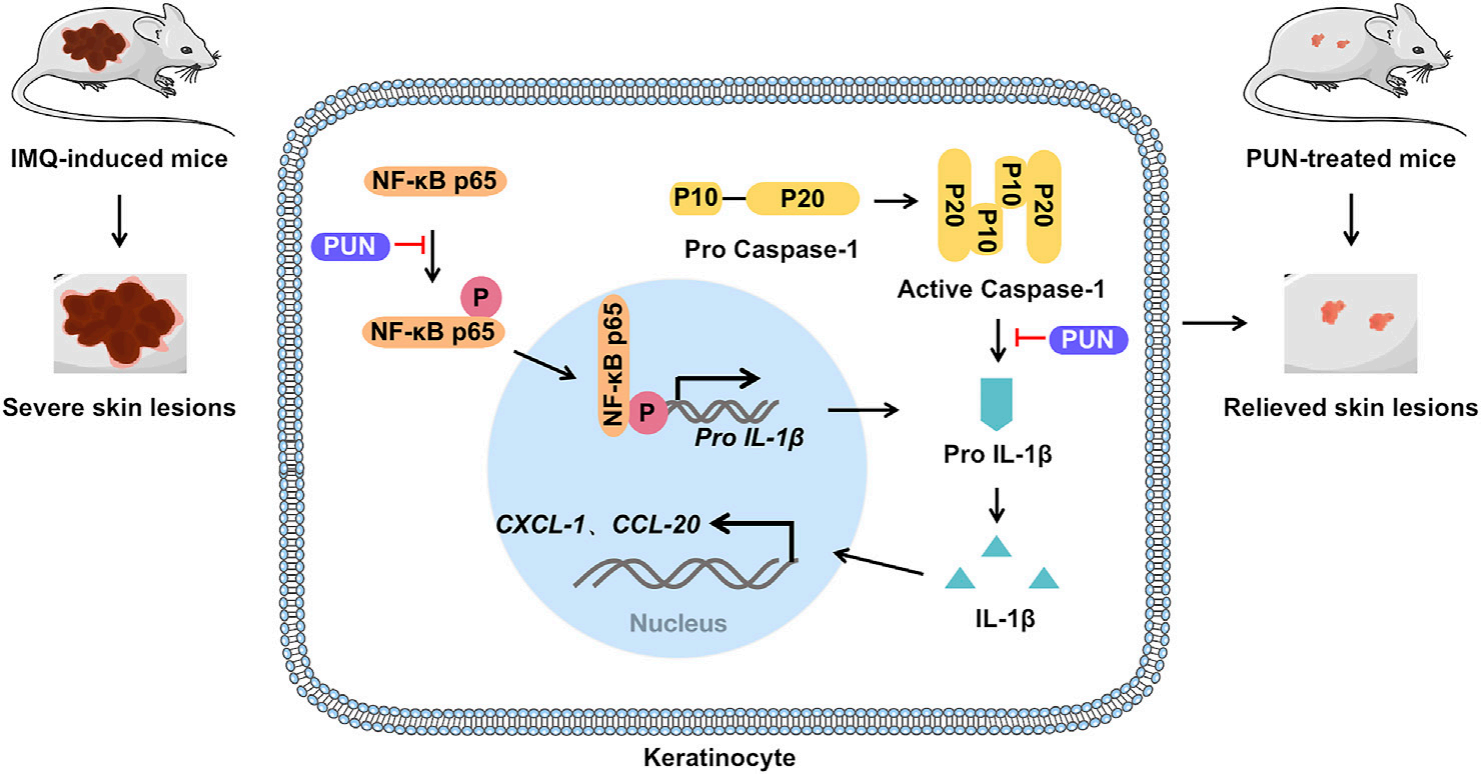
Punicalagin alleviates psoriasis by inhibiting NF-κB-mediated IL-1β transcription and caspase-1-regulated IL-1β secretion. Punicalagin relieves psoriasis by inhibiting NF-κB activation and cleaved caspase-1 expression, which ultimately suppresses the transcription and secretion of IL-1β and consequently represses IL-1β-mediated inflammatory cascade *in vitro* and *in vivo*.

PUN, the most abundant polyphenol in pomegranate (*Punica granatum L.*) peel, is often used in health care products or cosmetics because of its excellent antioxidant effects. Recently, PUN is proven to be capable of treating various diseases, including cervical cancer (Zhang et al., 2020), non-small cell lung malignancies (Fang et al., 2021), osteoarthritis (Liu F. et al., 2021) and diabetic cardiomyopathy (Fu et al., 2021). However, its effects on psoriasis remain unknown so far. Here, we gave the first evidence that PUN can alleviate the severity of psoriasis by downregulating IL-1β expression and subsequent IL-1β-mediated inflammatory responses *in vitro* and *in vivo* (Figure 4).

Previous studies demonstrated the key pathogenetic role of IL-1β in aggravating psoriatic inflammatory cascade and recalling psoriasis recurrent-related inflammatory memory (Naik et al., 2017; Cai et al., 2019b). Therefore, targeting IL-1β might be a promising strategy for treating psoriasis. Recently, Mansouri et al. (2015) and Skendros et al. (2017) independently showed that using IL-1β antagonists, such as canakinumab and gevokizumab, can successfully reduce the area and severity index scores of generalized pustular psoriasis. In this study, we present another evidence that targeting IL-1β by PUN could ameliorate psoriasis. These studies suggest that IL-1β inhibitors might open a novel therapeutic avenue for psoriasis.

Formerly, Shah et al. (2016) showed that PUN significantly decreased the gene expression of IL-1β. Besides the regulatory of PUN on the IL-1β gene expression, Huang et al. (2021) showed that PUN also reduces the protein level of IL-1β. Moreover, Martín-Sánchez et al. (2016) and Saeki et al. (2020) found that PUN affects the release of IL-1β by changing the membrane permeabilization. Consistent with these studies, we also found that PUN can regulate the expression and secretion of IL-1β through different pathways. On one hand, PUN can downregulate the transcription of IL-1β *via* suppressing NF-κB activation. On the other hand, PUN can also repress the maturation and secretion of IL-1β by inhibiting caspase-1-mediated pro-IL-1β cleavage. These studies indicate that PUN can function as a potent IL-1β inhibitor to treat various IL-1β-related diseases.

Although most psoriasis-associated studies have mainly focused on the imbalance of T helper 17 cells (Th17) and regulatory T cells (Treg), emerging studies indicate that keratinocytes also play a pivotal role in the initiation and the amplification of psoriasis (Greb et al., 2016). For example, Lande et al. (2014) showed that the antimicrobial peptide LL37, which is released from keratinocyte, can serve as an autoantigen to trigger the initial immune responses in psoriasis. Moreover, Hawkes and others proposed a feedback loop of “Dendritic cell—Th17 cell - Keratinocytes” in 2017 (Hawkes et al., 2017). In this “feed-forward” model, the activated myeloid dendritic cells produce IL-23 and IL-23 acts on Th17 cells to produce IL-17 cytokines. Then, these IL-17 cytokines (such as IL-17A) can stimulate keratinocytes to release a variety of cytokines, chemokines and antimicrobial peptides. These newly released antimicrobial peptides can further trigger the onset of psoriasis. In addition, the cytokines (such as IL-1β) and chemokines (such as CCL-20), which are released from keratinocytes upon IL-17A stimulation, can further amplify local inflammatory responses by increasing dermal IL-17 producing γδ T17 cell expansion. Taken together, these studies suggest that keratinocyte is a critical player in the pathogenesis of psoriasis (Ni and Lai, 2020). Given the important pathogenetic roles of keratinocytes in psoriasis, targeting keratinocyte might be a new therapeutic way for the treatment of psoriasis. Our studies showed that PUN can block this feedback loop of “Dendritic cell—Th17 cell—Keratinocytes” by downregulating the excessive expression of IL-1β and repressing the subsequent IL-1β-triggered inflammatory cascade in keratinocytes. Ultimately, this inhibition of “Dendritic cell—Th17 cell—Keratinocytes” feedback loop contributes to the pharmacological effects of PUN on psoriasis.

Recently, An et al. (2020) and Ge et al. (2021) reported that PUN can ameliorate diabetic nephropathy and collagen-induced arthritis by attenuating nucleotide-binding oligomerization domain, leucine-rich repeat and pyrin domain containing protein 3 (NLRP3)/caspase-1/gasdermin D (GSDMD)-mediated pyroptosis, respectively. Since that pyroptosis is a form of programmed cell death characterized by gasdermin family protein-mediated pore formation, cellular lysis and the release of IL-1β, Broz et al. (2020) and Liu X. et al. (2021) propose an idea that PUN is also a pyroptosis inhibitor. Given the pharmacological effects of PUN on psoriasis in our study, it will be really interesting to determine whether pyroptosis is also involved in the pathogenesis of psoriasis. The relation between pyroptosis and psoriasis may expand our understanding of pathogenesis of psoriasis.

In summary, we demonstrated that PUN alleviates psoriasis by suppressing excessive IL-1β expression and secretion. Mechanistically, functional inhibition of the NF-κB activation and cleaved caspase-1 expression contributes to the anti-psoriatic effects of PUN (Figure 4). Our findings suggest that punicalagin might be a promising therapeutic agent for the treatment of psoriasis in future.

## Data Availability

The raw data supporting the conclusion of this article will be made available by the authors, without undue reservation.
