# Supplementary figures and images for: Punicalagin Alleviates Psoriasis by Inhibiting NF-κB-Mediated IL-1β Transcription and Caspase-1-Regulated IL-1β Secretion
Source: Front Pharmacol. 2022 Jan 26;13:817526. doi: 10.3389/fphar.2022.817526 (PMC8826397; doi:10.3389/fphar.2022.817526)

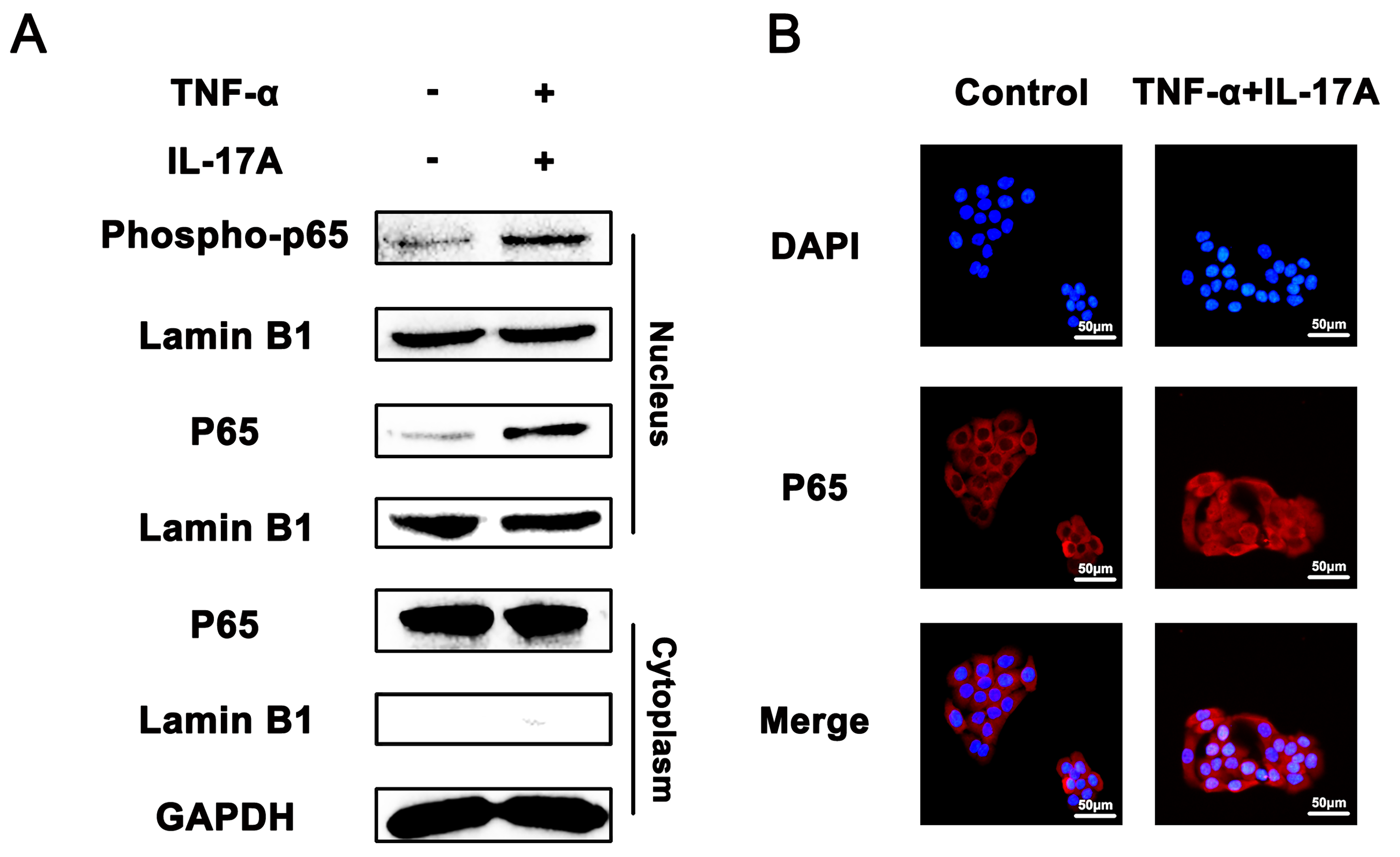

Supplement: Supplementary file 1 [file Image2.TIF]

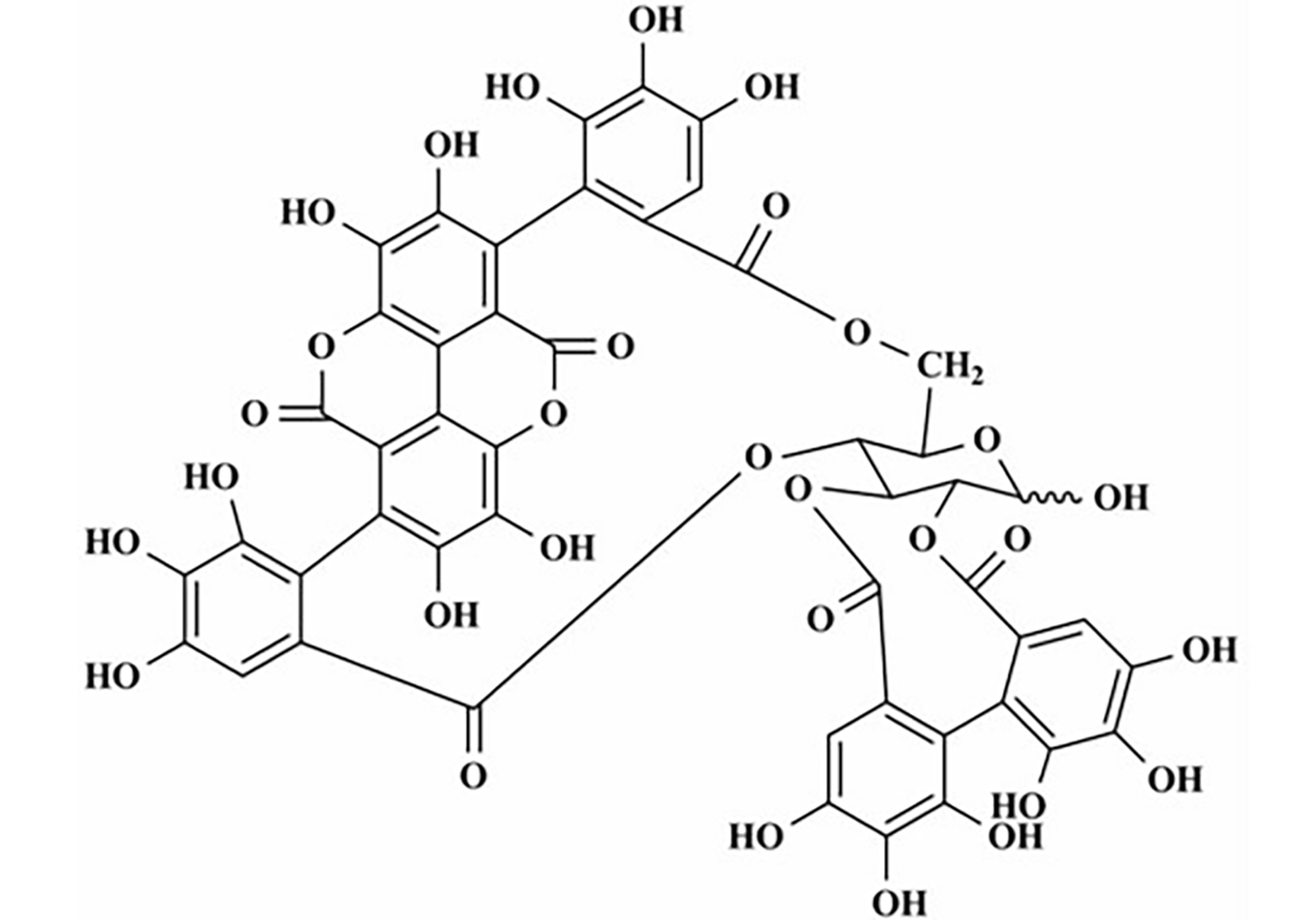

Supplement: Supplementary file 2 [file Image1.TIF]
