# Supplementary material for: Punicalagin Alleviates Psoriasis by Inhibiting NF-κB-Mediated IL-1β Transcription and Caspase-1-Regulated IL-1β Secretion
Source: Front Pharmacol. 2022 Jan 26;13:817526. doi: 10.3389/fphar.2022.817526 (PMC8826397; doi:10.3389/fphar.2022.817526)
Supplement: Supplementary file 3 [file DataSheet1.DOCX]

***Supplementary Materials***

# Supplementary Figures


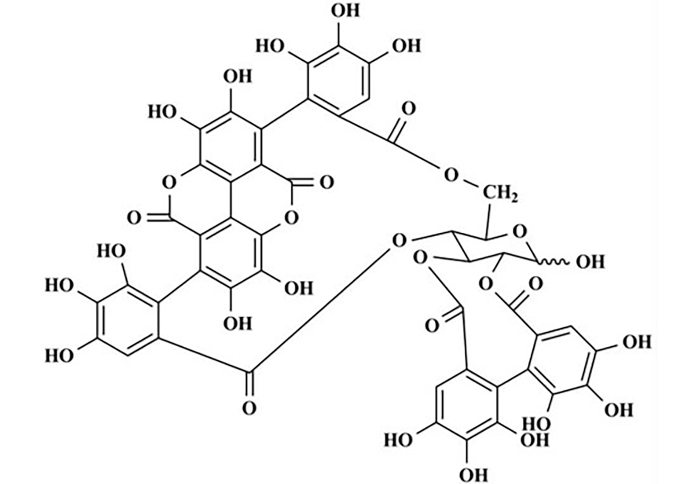


**Figure S1.** The structural formula of punicalagin


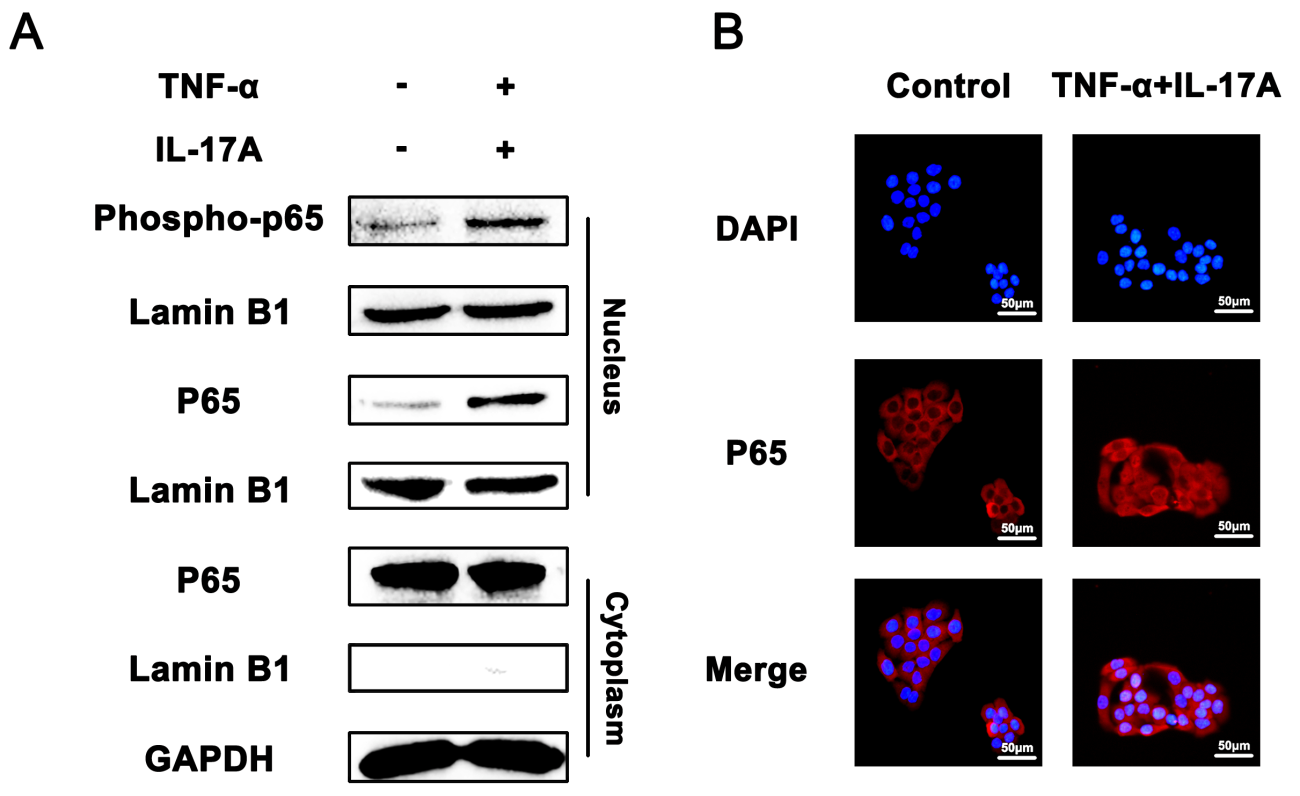


**Figure S2. NF-κB undergoes activation through phosphorylation of p65 (Ser536) and nuclear translocation of p65 after stimulation with IL17A (25ng/ml) and TNF-α (25ng/ml).** (A) The expression of phosphorylation (Ser536) and total p65 in the cytoplasm and nucleus after exposure of IL-17A and TNF-α for 24 hr. (B) HaCaT cells were incubated with TNF-α and IL-17A for 24 hr. Then, the location of p65 was detected by immunostaining technique. Scale bar, 50 μm.
